# Impact of minimal inhibitory concentration breakpoints on local cumulative bacterial susceptibility data and antibiotic consumption

**DOI:** 10.1186/1756-0500-7-603

**Published:** 2014-09-03

**Authors:** Sofia Stokkou, Ina Tammer, Stefanie Zibolka, Christina Grabau, Gernot Geginat

**Affiliations:** Institut für Medizinische Mikrobiologie und Krankenhaushygiene, Universitätsklinikum Magdeburg, Leipziger Str. 44, 39120 Magdeburg, Germany; Zentralapotheke, Universitätsklinikum Magdeburg, Leipziger Str. 44, 39120 Magdeburg, Germany

**Keywords:** Antibiotic susceptibility testing, Minimal inhibitory concentration, Clinical breakpoints, EUCAST, Antibiotic consumption, Antibiotic stewardship

## Abstract

**Background:**

The phenotypic antimicrobial susceptibility testing (AST) of bacteria depends on minimal inhibitory concentration breakpoints issued by national and international breakpoint committees. The current study was performed in order to test the influence of different AST standards on local cumulative AST data and on antibiotic consumption.

**Methods:**

Automated AST was performed with clinical isolates of *Pseudomonas aeruginosa, Escherichia coli, Klebsiella pneumoniae, Proteus mirabilis, Staphylococcus aureus,* coagulase-negative staphylococci, *Enterococcus faecalis*, and *E. faecium*. From each species 100 prospectively collected non-duplicate clinical isolates were tested and MIC data were interpreted according to the interpretation standards issued by DIN and EUCAST, respectively. In addition cumulative AST data from clinical isolates and antibiotic consumption were monitored before and after implementation of new EUCAST MIC breakpoints.

**Results:**

The susceptibility rate of *P. aeruginosa* against piperacillin and gentamicin, and of *C. freundii* against piperacillin/tazobactam increased significantly, whereas the susceptibility rates of *E. cloacae*, *S. marcescens*, and *M. morganii* against ciprofloxacin decreased significantly after switching from DIN to EUCAST MIC breakpoints. These changes in the cumulative antibiotic resistance pattern were reflected by enhanced consumption of piperacillin/tazobactam after implementation of EUCAST MIC breakpoints.

**Conclusions:**

These data show that changes of AST breakpoints have a significant influence on local cumulative AST data and on antibiotic consumption.

## Background

The phenotypic antimicrobial susceptibility testing (AST) of bacteria depends on minimal inhibitory concentration (MIC) breakpoints issued by national and international breakpoint committees. The most common MIC breakpoints used in Germany are DIN 2004 [1], EUCAST version 2.0 from 2011 [2], and CLSI 2010 [3].

The MIC breakpoints issued by DIN in 2004 and EUCAST in 2011 differ considerably (Table 1). To which extent different breakpoints influence the grouping of bacteria into the categories susceptible, intermediate, and resistance depends on the actual MIC distribution of isolates.

**Table 1:** **Species drug combinations tested and MIC breakpoints according to DIN 2004 and EUCAST 2.0**

	MIC breakpoints (μg/ml)					
	DIN			EUCAST		
Drug	S	I	R	S	I	R
*Enterobacteriaceae*						
pip./taz.	≤4	8-32	>32	≤8	16	>16
cefotaxime	≤2	4-8	>8	≤1	2	>2
imipenem^a^	≤2	4	>4	≤2	4-8	>8
ciprofloxacin	≤1	2	>2	≤0.5	1	>1
gentamicin	≤1	2-4	>4	≤2	4	>4
*Pseudomonas aeruginosa*						
piperacillin	≤4	8-32	>32	≤16	─	>16
ceftazidime	≤4	8-16	>16	≤8	─	>8
imipenem	≤2	4	>4	≤4	8	>8
meropenem	≤2	4-8	>8	≤2	4-8	>8
ciprofloxacin	≤1	2	>2	≤0.5	1	>1
gentamicin	≤1	2-4	>4	≤4	─	>4
*Stenotrophomonas maltophilia*						
trim./sulfam.	≤16	32-64	>64	≤4	─	>4
* Acinetobacter baumanii*						
imipenem	≤2	4	>4	≤2	4-8	>8
meropenem	≤2	4-8	>8	≤2	4-8	>8
ciprofloxacin	≤1	2	>2	≤1	─	>1
gentamicin	≤1	2-4	>4	≤4	─	>4
*Enterococcus spp.*						
ampicillin	≤2	4-8	>8	≤4	8	>8
vancomycin	≤4	8	>8	≤4	─	>4
teicoplanin	─	─	─	≤2	─	>2
linezolid	─	─	─	≤4	─	>4
*Staphylococcus spp.*						
oxacillin^b^	≤1	─	>1	≤2	─	>2
oxacillin^c^	≤1	─	>1	≤0.25	─	>0.25
vancomycin^b^	≤4	8	>8	≤2	─	>2
vancomycin^c^	≤4	8	>8	≤4	─	>4
teicoplanin^b^	─	─	─	≤2	─	>2
teicoplanin^c^	─	─	─	≤4	─	>4
linezolid	─	─	─	≤4	─	>4
rifampicin	─	─	─	≤0.06	─	>0.5
clindamycin	≤1	2-4	>4	≤0.25	0.5	>0.5

^a^Proteus, Morganella, and Providencia spp. were tested against meropenem, ^b^breakpoints for S. aureus, ^c^breakpoints for coagulase-negative staphylococci, “─” no data.

While EUCAST and CLSI mostly use species-specific breakpoints the DIN until 2010 relied on species-independent breakpoints only. EUCAST also provides species-independent MIC breakpoints, however, species-independent breakpoints for the reading of disk diffusion tests are not available. Species-independent breakpoints facilitate routine AST testing, especially of uncommon isolates for which often no species-specific breakpoints are available. This is one reason why DIN breakpoints are still frequently used in Germany. From AST results of bacterial isolates microbiology labs compile cumulative AST data summaries for hospitals that are intended to guide empirical antimicrobial therapy [4].

Despite DIN, CLSI, EUCAST, and various other breakpoints are widely used in Europe little is known how different MIC breakpoints influence cumulative AST data and antibiotic consumption. Therefore, in the process of switching from DIN to EUCAST MIC breakpoints we studied the influence of different breakpoints on cumulative AST data and monitored the effect of the implementation of EUCAST MIC breakpoints on antibiotic consumption.

## Methods

### Compilation of cumulative AST data

Before implementation of new MIC breakpoints the possible effects of different MIC breakpoints on cumulative AST data were evaluated by testing 100 consecutive isolates of frequently isolated clinically important bacteria such as *Pseudomonas aeruginosa*, *Escherichia coli*, *Klebsiella pneumoniae*, *Enterobacter cloacae, Proteus mirabilis*, *Staphylococcus aureus*, coagulase-negative staphylococci, *Enterococcus faecalis*, and *E. faecium* using DIN and EUCAST MIC breakpoints, respectively. In the time period available for the study we also collected consecutive isolates of less frequent species such as *Stenotrophomonas maltophilia* (n = 47), *Acinetobacter baumannii* (n = 27), *Serratia marcescens* (n = 37), *Morganella morganii* (n = 30) and *Citrobacter freundii* (n = 20) which were tested using DIN and EUCAST MIC breakpoints. In addition panels of 35 extended-spectrum-beta-lactamase (ESBL)-positive, fluoroquinolone-resistant Enterobacteriaceae, 26 multi-drug-resistant (MDR) *P. aeruginosa* [5], and 19 methicillin-resistant *S. aureus* (MRSA) isolates were included in the study.

From individual patients only the first isolate per analysis period was included in order to eliminate duplicate isolates. Identification of all isolates was performed by mass spectrometry (VITEK MS, Biomerieux, Nürtingen, Germany) and AST of all isolates was performed using the VITEK 2 (Biomerieux) with AST-N118, AST-N263, AST-P580, and AST-P586 panels, respectively. All isolates were collected as part of standard patient care and processed according to the standard protocols provided by the manufacturer. For AST of strain collections the VITEK 2 expert system was inactivated and thus no interpretative reading of AST was performed. Results were categorized according to DIN 2004 and EUCAST 2.0 (2011) VITEK 2 MIC breakpoints, respectively. The individual species-specific breakpoints for the clinical categories susceptible, intermediate, and resistant are provided in Table 1. From these data the frequencies of susceptible, resistant, and intermediate isolates were calculated. ESBL-confirmation tests were performed with ESBL CT/CTL, ESBL TZ/TZL, and ESBL PM/PML double E-tests (Biomerieux, [6]) according to the instructions provided by the manufacturer.

In addition cumulative AST data were compiled for all clinical isolates for the 12 months before implementation of EUCAST MIC breakpoints (quarters 3/2011 to 2/2012) and 12 months (quarters 3/2012 to 2/2013) after implementation of new breakpoints. Also for cumulative AST data from individual patients only the first isolate irrespective of body site was included in order to eliminate duplicates. The AST of routine clinical isolates was performed with active VITEK 2 expert system and thus interpretative reading of AST results was performed.

### Antibiotic consumption

Antibiotic consumption was determined for 12 months (quarters 3/2011 to 2/2012) before and 12 months (quarters 3/2012 to 2/2013) after the implementation of EUCAST MIC breakpoints. From the prescription data of the hospital pharmacy the numbers of defined daily doses according to WHO standards per 1000 patient-days were calculated [7].

### Statistical analysis

Only bacteria/drug combinations with a MIC breakpoint-dependent variation of the bacterial susceptibility rate equal to or higher than 10% were analyzed. This threshold was used in order to focus on potentially clinical relevant changes in bacterial susceptibility rates. For statistical analysis of cumulative antibiotic resistance data 2-way contingency tables (sensitive isolates versus the sum of intermediate and resistance isolates) were tested using the chi-square test at the p < 0.05 significance level. Statistical analysis of antibiotic consumption data was performed using the distribution-free Mann–Whitney *U*-test at the p < 0.05 significance level.

### Ethics statement

According to the written decision of the clinical research ethics committee of the University of Magdeburg the current study did not require approval by the local ethics committee because no human material or data attributable to individual patients were used.

## Results

### Influence of MIC breakpoints on cumulative AST data compiled from strain collections

In order to predict the potential changes in cumulative AST data before implementation of new EUCAST MIC breakpoints collections of clinically important bacteria were tested according to DIN and EUCAST MIC breakpoints.

Table 2 provides the cumulative AST data for clinically important species/drug combinations categorized into susceptible, intermediate, and resistant according to DIN and EUCAST MIC breakpoints, respectively. The comparison of the cumulative susceptibility data showed a statistically significant variation in the category susceptible of at least 10% in 5 species/drug combinations. The strongest effect on the susceptibility rate was observed in the combination *P. aeruginosa*/piperacillin. The susceptibility rate of *P. aeruginosa* against piperacillin was more than 50% higher according to EUCAST compared to DIN MIC breakpoints. The rates of piperacillin/tazobactam-susceptible isolates of *K. pneumoniae* and of gentamicin-susceptible *P. aeruginosa* were 12% and 22% higher according to EUCAST compared to DIN MIC breakpoints (Table 2). Among the tested species/carbapenem combinations no changes of the susceptibility rates ≥ 10% were observed. The observed changes of the susceptibility rates against ciprofloxacin were only significant with *P. aeruginosa* and *M. morganii* that according to EUCAST yielded 12% and 13% lower susceptibility rates against ciprofloxacin compared to DIN, respectively. The susceptibility against 3rd generation cephalosporins was not significantly affected by the application of different interpretative criteria.

**Table 2:** **Species drug combinations with changes ≥ 10% in the category susceptible in cumulative AST data prepared form representative strain collections**

	Assignment of strain collections to AST categories (%)					
	DIN			EUCAST ^*^		
***Species*** ^#^/drug	S	I	R	S	I	R
*K. pneumoniae* (n = 100)						
pip./taz.	72	24	4	84^*^	9	7
*P. mirabilis* (n = 100)						
ciprofloxacin	78	15	7	68	10	22
*M. morganii* (n = 30)						
cefotaxime	83	13	3	73	10	17
ciprofloxacin	100	0	0	87^*^	13	0
*P. aeruginosa* (n = 100)						
piperacillin	40	56	4	91^*^	0	9
ciprofloxacin	88	5	7	76^*^	12	12
gentamicin	73	22	5	95^*^	0	5

^*^Asterisks indicate a significant (p < 0.05) difference of the number of susceptible isolates according to EUCAST MIC breakpoints compared to DIN.

^#^No variation ≥ 10% in the category susceptible was found for *E. coli* (n = 100), *E. cloacae* (n = 100), *S. marcescens* (n = 37), *C. freundii* (n = 20), *S. maltophilia* (n = 47), *S. aureus* (n = 100), coagulase negative staphylococci (n = 100), *E. feacalis* (n = 100), and *E. faecium* (n = 100).

As highly resistant isolates were not well represented in the collection of 100 consecutive clinical isolates we additionally tested panels of 35 ESBL-positive, fluoroquinolone-resistant Enterobacteriaceae, 26 MDR *P. aeruginosa* [5], and 19 MRSA isolates, respectively (Table 3). In contrast to the data obtained with the unselected *E. coli* isolates (Table 2) the analysis of ESBL-producing *E. coli* showed a significantly enhanced, 29% higher susceptibility rate against piperacillin/tazobactam if EUCAST instead of DIN MIC breakpoints were applied.

**Table 3:** **Effect of AST breakpoint on cumulative AST data prepared form representative strain collections of ESBL-expressing** ***E. coli*** **, MDR** ***P. aeruginosa*** **, and MRSA isolates**

	Assignment of strain collections to AST categories (%)					
	DIN					
***Species***/drug	S	I	R	S	I	R
*E. coli* ESBL (n = 35)						
pip./taz.	51	31	17	80^*^	6	14
MDR *P. aeruginosa* (n = 26)						
piperacillin	0	42	58	15^*^	0	85
ceftazidime	38	31	31	50	0	50
ciprofloxacin	27	15	58	8	19	73
gentamicin	42	19	38	62	0	38

*MRSA* (n = 19) - no variation ≥ 10% in the category susceptible.

^*^Asterisks indicate a significant (p < 0.05) difference of the number of susceptible isolates according to EUCAST MIC breakpoints compared to DIN.

The AST of MDR *P. aeruginosa* isolates revealed similar trends as the testing of unselected *P. aeruginosa* isolates. Testing with piperacillin, ceftazidime, and gentamicin yielded a trend to enhanced susceptibility rates while the combination *P. aeruginosa*/ciprofloxacin showed a trend to lower susceptibility rates (Table 3). Due to the low number of strains tested, however, this trend was statistically significant for the test combination *P. aeruginosa*/piperacillin only.

### Changes of hospital cumulative AST data after implementation of EUCAST MIC breakpoints

In the process of implementing new EUCAST MIC breakpoints cumulative AST data were compiled for the periods of 12 month before and 12 month after implementation of the new MIC breakpoints. Table 4 summarizes the six species/drug combinations which showed a change of the susceptibility rate equal to or larger than 10%. We focused on changes of at least 10% because these changes are of potential clinical interest. Only gram-negative bacteria were affected. With an increase of the susceptibility rate of 40% the combination *P.aeruginosa*/piperacillin showed the highest change of the susceptibility rate of all species/drug combinations tested. The second strongest effect was the enhancement of the susceptibility rate of *P. aeruginosa* against gentamicin from 55% to 92%.

**Table 4:** **Species/drug combinations with changes ≥ 10% in the category susceptible after implementation of EUCAST MIC breakpoints**

	Assignment of clinical isolates to AST categories (%)					
	Q3 2011-Q2 2012 (DIN)			Q3 2012-Q2 2013 ^*^ (EUCAST)		
***Species*** ^#^/drug	S	I	R	S	I	R
*E. cloacae*	n = 77				n = 41	
ciprofloxacin	97	0	3	85^*^	9	6
*S. marcescens*	n = 116				n = 93	
ciprofloxacin	99	0	1	88^*^	9	3
*M. morganii*	n = 107			n = 92		
ciprofloxacin	97	0	3	82^*^	10	8
*C. freundii*	n = 93			n = 89		
pip/taz	77	4	19	87^*^	4	9
*P. aeruginosa*	n = 462			n = 444		
piperacillin	41	53	7	81^*^	3	16
gentamicin	55	36	9	92^*^	0	8

^*^Asterisks indicate a significant (p < 0.05) of the number of susceptible isolates according to EUCAST MIC breakpoints compared to DIN.

^#^No variation ≥ 10% in the category susceptible was found for *E. coli*, *K. pneumoniae*, *P. mirabilis*, *S. maltophilia*, *A. baumannii*, *S. aureus*, coagulase negative staphylococci, *E. feacalis*, and *E. faecium*.

The testing of *E. cloacae*, *S. marcescens*, and *M. morganii* against ciprofloxacin showed significant reductions of the ciprofloxacin susceptibility rates of 12%, 11%, and 15%, respectively. Among the Enterobacteriaceae the only other species/drug combination affected was *C. freundii*/piperacillin/tazobactam which showed a significant increase of the susceptibility rate of 10% after implementation of EUCAST MIC breakpoints.

### Changes of hospital antibiotic consumption after implementation of EUCAST MIC breakpoints

**Figure 1:**
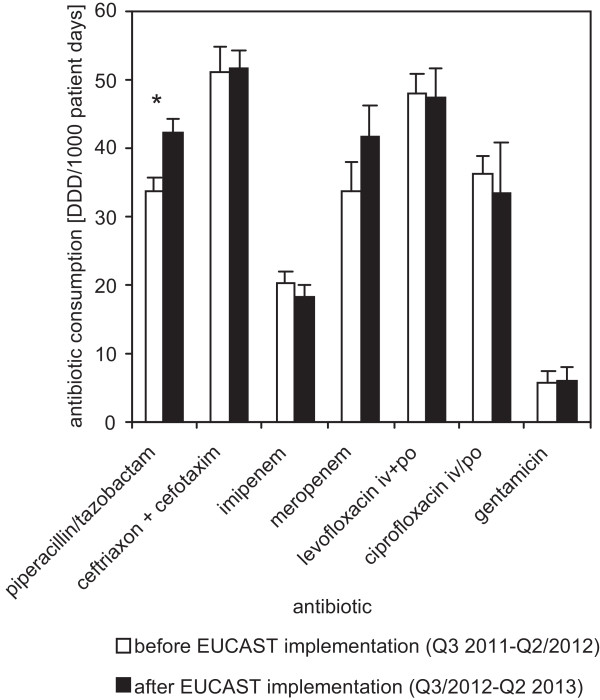
**Antibiotic consumption of selected antibiotics before and after implementation of EUCAST MIC breakpoints.** The prescribed defined daily doses per 1000 patient days was calculated for the study periods of 12 month before (open bars) and 12 month after (closed bars) implementation of EUCAST MIC breakpoints. Indicated are the means and standard deviations of the 4 quarters before and the 4 quarters after implementation of EUCAST MIC breakpoints. The asterisk indicates a statistically significant (p < 0.05) change of antibiotic consumption.

In order to evaluate if theses changes of cumulative hospital AST data had any effects on antibiotic prescription the numbers of prescribed defined daily doses of piperacillin/tazobactam, ceftriaxone + cefotaxime, meropenem, imipenem, ciprofloxacin iv/po, levofloxacin iv/po, and gentamicin per 1000 patient days was calculated for the periods of 12 month before and after implementation of EUCAST MIC breakpoints (Figure 1). The direct comparison of these two periods shows that the prescription per patient day of piperacillin/tazobactam significantly (p < 0.05) increased after implementation of EUCAST MIC breakpoints. No significant change in antibiotic consumption occurred among the other antibiotics monitored.

## Discussion

Our data show that changes in clinical breakpoints affect cumulative antibiotic susceptibility data and antibiotic consumption. To our knowledge, this is the first study that demonstrates a shift of antibiotic consumption after switching of MIC breakpoints.

The effect of any change in clinical breakpoints depends on the MIC distribution of the isolates analyzed. In the current study, applying both DIN and EUCAST MIC breakpoints to a panel of MDR *P. aeruginosa* isolates revealed a 15% increase in susceptibility against piperacillin when current EUCAST instead of DIN 2004 clinical breakpoints are applied. In contrast to this rather limited effect of MIC breakpoints on cumulative AST data of selected MDR *P. aeruginosa* isolates a more dramatic effect was observed in the panel of 100 consecutive *P. aeruginosa* isolates. If EUCAST MIC breakpoints were applied the susceptibility rate of *P. aeruginosa* in comparison to DIN increased from 40% to greater than 90%. The analysis of the collection of 100 consecutive *P. aeruginosa* strains quite precisely predicted the actual change of the susceptibility rate of *P. aeruginosa* after implementation of EUCAST breakpoints which increased from 41% to 81%. Remarkably, the enhanced susceptibility rate of piperacillin/tazobactam correlated with increased consumption of this antibiotic.

In contrast to piperacillin/tazobactam, the enhanced susceptibility of *P. aeruginosa* against gentamicin did not result in increased gentamicin consumption. A probable explanation is that because at our institution combination therapy with gentamicin is mostly used for the treatment of endocarditis and only rarely for the treatment of severe *P. aeruginosa* infections the increased susceptibility against gentamicin resulted in very few additional treatments with gentamicin.

Other changes of cumulative AST data after introduction of EUCAST MIC breakpoints that are of potential clinical relevance are the reduced susceptibility of *P. aeruginosa*, *P. mirabilis*, and *M. morganii* against ciprofloxacin. EUCAST MIC breakpoints of fluoroquinolones for Enterobacteriaceae and *P. aeruginosa* are lower than DIN or CLSI 2012 MIC breakpoints. Resistance against fluoroquinolones is a multistep process which involves multiple mutations in genes coding for bacterial DNA gyrase and topoisomerase IV [8]. Thus when using EUCAST MIC breakpoints low level fluoroquinolone resistance is detected in some isolates that appear still susceptible according to CLSI 2012 or DIN clinical breakpoints resulting in significantly decreased susceptibility of various Enterobacteriaceae against fluorquinolones. This change in fluorquinolone resistance of Enterobacteriaceae, however, did not result in significantly reduced hospital consumption of fluorquinolones.

A number of previous studies [9–14] reported the influence of different MIC breakpoints on cumulative AST data. The current study differs from these studies i) by comparing different breakpoint systems (DIN vs. EUCAST), ii) by studying both strain collections as well as hospital cumulative AST data collected before and after implementation of new breakpoints, and iii) by assessing changes of hospital antibiotic consumption after introduction of new EUCAST breakpoints.

Neither current EUCAST nor CLSI guidelines recommend the editing of susceptible and intermediate AST results of ESBL-producing Enterobacteriaceae against penicillins and cephalosporins into resistant. This reporting policy of AST-results of ESBL- and AmpC-producing bacteria is still the matter of debate [15–17] and thus many institutions, still test for the presence of ESBL-production. In the current analysis editing for ESBL-producing strains was performed for routine AST while the comparison of AST data from strain collections was performed without editing. Thus ESBL editing explains why ESBL-*E.coli* and *K. pneumoniae* were more suceptible to piperacillin/tazobactam in the analysis based on strain collections compared to cumulative hospital AST data.

The differences of DIN and EUCAST MIC breakpoints for Enterobacteriaceae and cefotaxime, however, resulted only in minor changes of cumulative AST data. Thus our data corroborate previous studies which reported no major differences in the susceptibility rates of ESBL-producing Enterobacteriaceae against cefotaxime if EUCAST and CLSI breakpoints were compared [10, 14]. Without interpretative reading and editing, however, cefotaxime resistance due to ESBL- or AmpC-expression by Enterobacteriaceae does not correlate with resistance against piperacillin/tazobactam [18]. In our study this was observed with ESBL-producing *E. coli* that according to EUCAST MIC breakpoints were > 80% susceptible against piperacillin/tazobactam despite producing ESBL.

It should be noted that the narrowing or elimination of the intermediate AST category in EUCAST MIC breakpoints may result in an increased rate of major errors (false resistant) and very major errors (false susceptible) [19]. This may be a particular problem for AST of *P. aerugiosa* against piperacillin a species/drug combination in which high numbers of major and very major errors occurred with automated AST [20, 21]. Thus it would be interesting to know how increased prescription of piperacillin/tazobactam correlated with the outcome of treated patients.

An important limitation of the current study is the lack of a control group which was monitored over the same time period without changing of MIC breakpoints. Such a control group, however, would require a multi centric approach which was beyond the scope of the current study. In our hospital internal guidelines for antibiotic prescription were introduced at the end of the 3rd quartal after implementation of new EUCAST guidelines and thus could have affected antibiotic prescription habits toward the end of the second monitoring period. Antibiotic consumption, however, did not change significantly in the quarter after introduction (data not shown). Nevertheless we can not exclude that the increased consumption of piperacillin/tazobactam after implementation of EUCAST breakpoints was influenced by other factors such as new publications, new guidelines, or enhanced promotional activities of pharmaceutical companies.

## Conclusions

Our data show that changes of AST breakpoints can significantly influence hospital cumulative AST data and antibiotic consumption. These effects have to be taken into consideration if local AST breakpoints are changed.

## Abbreviations

AST: Antimicrobial susceptibility testing
CLSI: Clinical Laboratory Standards Institute
DIN: Deutsches Institut für Normung
ESBL: Extended-spectrum beta-lactamase
EUCAST: European Committee on Antimicrobial Susceptibility Testing
MDR: Multi-drug-resistant
MIC: Minimal inhibitory concentration
MRSA: Methicillin-resistant *Staphylococcus aureus.*
